# A comparative study of pitch recognition in children with cochlear implants and normal hearing peers across Mandarin tones

**DOI:** 10.3389/fpsyg.2026.1783243

**Published:** 2026-07-02

**Authors:** Yishun Gu, Fuhai An, Shengnan Ge, Qiang Guo, Minmin Yin

**Affiliations:** 1 Jing Hengyi School of Education, Hangzhou Normal University, Hangzhou, China; 2 Department of Rehabilitation Medicine, Huashan Hospital, Fudan University, Shanghai, China; 3 College of Child Development and Education, Zhejiang Normal University, Hangzhou, China; 4 Zhejiang Philosophy and Social Science Laboratory for Research in Early Development and Childcare, Hangzhou Normal University, Hangzhou, China

**Keywords:** auditory perception, cochlear implantation, Mandarin tones, normal-hearing children, pitch recognition

## Abstract

**Objective:**

This study aims to systematically compare the pitch recognition abilities of Mandarin-speaking children with cochlear implants (CI) and their normal-hearing (NH) peers, examining performance variations across different pitch levels (high, normal, low) and Mandarin lexical tones (T1–T4).

**Methods:**

A comparative experiment was conducted involving 25 children with CI and 25 age-matched NH children. Using acoustic analysis, pitch recognition was assessed through a task where participants identified pitch levels in sentences representing the four Mandarin tones.

**Results:**

Children with CI demonstrated a dissociated pattern of pitch recognition. Their performance in recognizing high pitch was comparable to that of NH children. However, they exhibited significant deficits in recognizing both normal and low pitch. Furthermore, the type of Mandarin lexical tone did not significantly modulate pitch recognition accuracy in either group.

**Conclusion:**

The findings indicate a selective deficit in pitch recognition among children with CI, primarily affecting the normal and low pitch ranges. The results provide empirical evidence for developing targeted auditory rehabilitation strategies that address the specific pitch recognition challenges faced by pediatric CI users.

## Introduction

Cochlear implants (CI) are the primary intervention for auditory rehabilitation in individuals with severe to profound sensorineural hearing loss, enabling many recipients to develop oral speech and language skills (Clark, 1998). However, despite significant technological advancements, a persistent challenge for CI users lies in the accurate perception of pitch. Pitch, defined as the subjective perceptual experience or auditory percept derived from fundamental frequency (F_0_), is crucial for understanding speech prosody—encompassing rhythm, stress, and intonation (Borrie et al., 2017)—and is fundamental for communication and social interaction.

The technological limitations of current CI are central to this difficulty. CI sound processing involves filtering the acoustic signal into frequency bands and extracting the temporal envelope to electrically stimulate the auditory nerve. This process, while effective for conveying speech in quiet, significantly degrades the spectrally fine structure and precise F_0_ information—the primary acoustic cue for pitch— that are essential for pitch perception (Fu and Shannon, 1999). As a result, CI users rely heavily on temporal envelope cues to perceive pitch. However, contrary to the intuitive expectation that pitch perception might be better for higher frequencies, Chatterjee and Peng (2008) demonstrated that temporal envelope cues provide more effective F_0_ representation at lower frequency ranges, and that CI users’ pitch perception accuracy systematically degrades as F_0_ increases. Consequently, children with CI often exhibit deficits in recognizing pitch variations, which can negatively impact their appreciation of speech prosody, music, and, critically for Mandarin-speaking children, lexical tone.

Mandarin Chinese is a tonal language where variations in pitch—arising from changes in F_0_— at the syllable level distinguish word meaning. The four lexical tones—Tone 1 (high-level), Tone 2 (mid-rising), Tone 3 (dipping), and Tone 4 (high-falling)—assign different meanings to the same syllable. It is crucial to distinguish between three levels of analysis here: F_0_ is a physical acoustic property of the speech signal; pitch is the perceptual sensation evoked by F_0_; and lexical tone is a phonemic category that conveys meaning and is acoustically realized through, and perceptually dependent on, pitch patterns. In other words, lexical tones are phonological categories realized acoustically through specific F_0_ contours and perceived by listeners as patterns of pitch variation. Thus, pitch serves as the primary perceptual cue through which lexical tone is recognized. Thus, for prelingually deafened children acquiring Mandarin, accurate pitch perception is not merely a prosodic skill but a fundamental requirement for lexical development and effective communication (Zhang et al., 2023). Given the degraded F_0_ information transmitted by CI, Mandarin-speaking children with CI face a dual challenge: they must not only process pitch for its prosodic function but also for its critical linguistic function in accessing tonal contrasts.

This challenge is compounded by the nature of the typical auditory environment. In early childhood, speech directed to infants and toddlers, known as child-directed speech (CDS), is characterized by exaggerated prosodic features, including a higher overall mean F_0_, wider F_0_ range, and slower tempo compared to adult-directed speech (ADS) (Fernald et al., 1989). These acoustic modifications are believed to attract attention and facilitate early language learning (Song et al., 2010). However, for children with CI, the very acoustic F_0_ cues that make CDS salient are those most poorly encoded by their device. Research confirms that while mothers of children with CI adjust their speech (Bergeson et al., 2006), their children’s perception of pitch and melody remains constrained (Huang et al., 2017).

A growing body of research has documented pitch recognition deficits in pediatric CI users. Nakata et al. (2012) found that children with CI show significant difficulties in both the perception and production of speech prosody, including the ability to discern emotional intonation. Studies have shown that children with CI have more difficulty discriminating pitch direction (See et al., 2013) and identifying lexical tones compared to their normal-hearing (NH) peers (Ciocca et al., 2002). These perceptual deficits can have cascading effects on broader linguistic and indexical speech skills, including the recognition of talker identity and emotional prosody (Geers et al., 2013). In Mandarin-speaking children with CI, this manifests as poorer overall tone identification, with particular difficulty distinguishing between Tone 2 and Tone 3, which share similar dynamic F_0_ contours (Chen et al., 2014; Li et al., 2016; Liu and Samuel, 2004). While these findings confirm a deficit, they do not clearly dissociate whether the observed difficulties stem primarily from the low-level auditory processing constraints affecting the encoding of F_0_ cues or from higher-level interactions with the phonological function of tone categories. Given the dual role of F_0_-based pitch in Mandarin—serving both linguistic (tonal) and paralinguistic (prosodic) functions—it remains unclear whether the pitch recognition difficulties observed in children with CI are uniformly applied across different linguistic contexts or are modulated by them.

Therefore, the primary rationale for this study is to disentangle the effects of device-imposed auditory constraints from linguistic function on pitch recognition in Mandarin-speaking children with CI. To achieve this, the present study aims to: (1) systematically compare pitch recognition abilities across different F_0_ levels (high, normal, low) between Mandarin-speaking children with CI and their age-matched NH peers; and (2) examine whether Mandarin lexical tone type modulates pitch recognition performance in either group.

In this study, we operationally defined and compared three pitch levels: “Normal,” “High,” and “Low.” The “Normal” pitch was designed to simulate the typical, neutral speaking voice of an adult female in a calm, declarative context, serving as an acoustic baseline. The “High” pitch was designed to mimic the elevated pitch register characteristic of CDS, which is known to be more attention-grabbing and perceptually salient. The “Low” pitch was included to test perception of a register below the typical conversational range, exploring the boundaries of pitch recognition. This definition directly links the experimental manipulation of pitch level to real-world speech registers discussed in the literature, thereby solidifying the conceptual framework.

By examining the underlying pitch recognition abilities that subserve tone recognition, this study aims to clarify whether observed deficits reflect general auditory processing limitations or interactions with phonological knowledge. Based on the established literature on CI limitations, we hypothesized that: (H1) Children with CI would show significantly poorer overall pitch recognition compared to NH children. (H2) We predicted a dissociated pattern of deficit, with the performance gap between children with CI and NH children being larger for normal and low pitch compared to high pitch. This exploratory prediction was based on two considerations: (a) the acoustically exaggerated, high-register speech (simulating CDS) may provide more salient amplitude modulation cues that are transmitted with reasonable fidelity by CI, potentially supporting pitch discrimination in that condition (Xu and Pfingst, 2008); and (b) the discrimination of normal and low pitch relies more critically on fine spectro-temporal details (i.e., temporal fine structure), for which CI provides a severely degraded representation (Lorenzi et al., 2006). We acknowledge that high F_0_ can also pose challenges due to spectral encoding limitations; this hypothesis tests the net outcome of these competing factors in our specific task. (H3) Lexical tone type would not significantly affect pitch recognition accuracy within the CI group, suggesting that the primary deficit lies in low-level auditory processing of F_0_ cues rather than in phonological categorization.

## Methods

### Participants

The study included two groups of child participants: children with CI and NH children. The inclusion criteria for each group were as follows:

In this study, 25 children with CI were selected from Hangzhou Wenhui School, Zhejiang Provincial Rehabilitation Medical Center, and Xiaoxiaohu Kindergarten in Jing’an District, Shanghai (Table 1). The inclusion criteria specified a chronological age between 3 and 6 years at the time of recruitment. The final sample of 25 children with CI had a chronological age at testing ranging from 3.5 to 6.5 years (mean = 5.09 ± 0.70 years). Their age at implantation ranged from 1.9 to 5.5 years, with a mean age of 3.65 ± 1.10 years. Among them, 12 were males and 13 were females. The inclusion criteria for children with CI were as follows: (1) Participants were aged between 3 and 6 years; (2) They had undergone CI (unilateral or bilateral) for at least 1 year, with the reconstructed hearing threshold reaching the appropriate level or higher; (3) They had pre-lingual deafness, with a recovery period of at least 6 months and some level of auditory perception; The minimum six-month post-implantation criterion was set to ensure a basic period of device acclimatization and reliable sound detection, while also intentionally capturing children in the early, dynamic phase of auditory (re)habilitation following their implant switch-on; (4) Mandarin was the primary language spoken, and participants were able to comprehend expressions of up to seven words; This language criterion was a practical, inclusive threshold designed to ensure that participants could understand the task instructions and the simple sentence stimuli used in the experiment, reflecting a minimum functional level required for testing. Standardized, comprehensive language test scores were not available for all participants beyond this functional criterion; and (5) Participants had no intellectual or other disorders, and were free from colds, middle ear inflammation, or other diseases, ensuring good physical condition during testing. This CI group comprised children with different hearing configurations: unilateral CI only (*n* = 15), bilateral CI (*n* = 6), and bimodal fitting (CI + hearing aid, *n* = 4). To reflect their real-world auditory experience, all children were tested in their everyday listening configuration (i.e., unilateral users with their CI only, bilateral users with both CI, and bimodal users with both devices). No device settings or fitting parameters were altered for the purpose of the experiment. Due to the limited sample size within each subgroup, hearing configuration was not included as a separate factor in the main statistical analysis.

**Table 1:** Specific situation of children with CI.

Serial number	Gender	Chronological age at testing (years)	Side	Starting time	Reconstruction effect	Recovery time
1	Female	3.5	Right	2022.09	Optimum	7 months
2	Male	3.75	Left	2022.02	Optimum	9 months
3	Male	4.25	Both	2020.09	Optimum	12 months
4	Female	4.33	Left	2021.06	Optimum	10 months
5	Male	4.5	Both	2020.05	Optimum	7 months
6	Male	4.75	Right	2020.04	Optimum	12 months
7	Female	4.67	Right	2021.05	Optimum	10 months
8	Female	4.75	Right	2021.08	Optimum	22 months
9	Female	5	Both	2020.12	Optimum	12 months
10	Female	5	Both	2019.11	Optimum	22 months
11	Female	5.33	Both	2020.10	Optimum	6 months
12	Male	5.42	Left	2021.02	Optimum	22 months
13	Male	5.5	Both	2020.09	Optimum	24 months
14	Female	5.67	Right	2022.12	Optimum	18 months
15	Male	5.75	Both	2018.10	Optimum	22 months
16	Female	5.75	Right	2018.10	Optimum	22 months
17	Male	5.83	Right	2018.05	Optimum	34 months
18	Female	5.92	Both	2018.12	Optimum	22 months
19	Male	5.08	Right	2022.06	Optimum	36 months
20	Male	4.92	Both	2020.05	Suit	39 months
21	Male	5.33	Right	2020.06	Optimum	36 months
22	Female	4.83	Both	2020.04	Optimum	30 months
23	Female	4.83	Right	2021.06	Optimum	26 months
24	Female	6	Left	2018.11	Optimum	24 months
25	Male	6.5	Left	2019.07	Optimum	36 months

In this study, 25 NH children were selected from the Future Science and Technology City Kindergarten, affiliated with Hangzhou Normal University, with age and gender matched to the children with CI. To ensure comparability, groups were matched based on their chronological age at the time of behavioral testing. An independent samples *t*-test confirmed no significant difference in age between the two groups [t(48) = 0.24, *p* = 0.812]. The NH participants’ ages ranged from 4.08 to 6.0 years, with a mean age of 5.11 ± 0.60 years. Of the participants, 12 were male and 13 were female. The inclusion criteria for NH children were as follows: (1) Participants were aged between 3 and 6 years; (2) Their hearing was normal, with the average hearing threshold of both ears being less than 25 dB HL; (3) Mandarin was the primary language spoken, and their language development was within normal limits; (4) They had no intellectual or developmental disorders, and no other systemic diseases or additional disorders; and (5) Participants had good physical and psychological conditions during testing, and were free from colds, middle ear inflammation, or other diseases.

It is important to note that criterion (3) for the CI group specified a minimum of 6 months of CI experience. As this period may be considered a relatively short duration for auditory acclimatization and perceptual learning, particularly in young children, the potential influence of limited CI experience, as well as the broader implications of the sample’s language abilities and developmental stage, on the results is also considered in the discussion section.

### Corpus compilation

#### Preparation of test materials

Given that the participants in this study were aged between 3 and 6 years, and considering the particular language and cognitive development characteristics of preschool children, the following principles were followed in preparing the test materials: (1) The test materials were semantically neutral, without any specific emotional tendencies; (2) The test materials were primarily composed of simple sentences with subject-predicate-object structures, each sentence containing five words, which aligns with the language development characteristics of preschool children; (3) The words used were common in daily life, and the content was simple and easy to understand, consistent with the cognitive development characteristics of preschool children. Additionally, this study was conducted within the context of Mandarin Chinese, and therefore incorporated four distinct tones (level tone, rising tone, falling-rising tone, and falling tone). Two sentences were selected for each of the four tones, along with two sentences featuring mixed tones. These 10 sentences were recorded using normal pitch, high pitch, and low pitch, resulting in a total of 30 sentences (as shown in Table 2).

**Table 2:** Test corpus of different tones.

Tone	Test material
T1	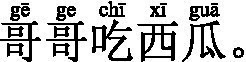 Brother eats watermelon. 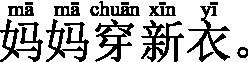 Mother wears a new dress.
T2	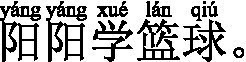 Yangyang learns basketball. 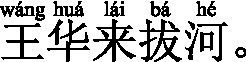 Wang Hua comes to tug-of-war.
T3	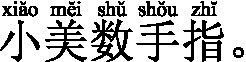 Xiao Mei counts her fingers. 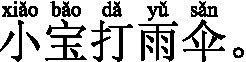 Xiao Bao holds an umbrella.
T4	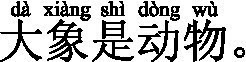 Elephants are animals. 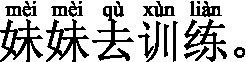 Sister goes to training.
Mixed-T	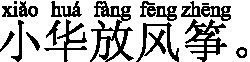 Xiao Hua flies a kite. 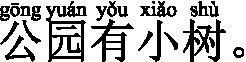 The park has small trees.

#### Recording and processing of corpus

The compiled corpus was recorded by a 36-year-old female adult who holds a second-level, first-class qualification in Mandarin. Mothers, who are typically the primary caregivers of children, were considered in the selection of the speaker. Additionally, data from the Ministry of Education in 2021 indicate that there are 2,848,600 female full-time preschool teachers in China, accounting for 97.78% of the total number of full-time preschool teachers. Given that female teachers constitute the primary workforce in preschool education, recordings made by a female speaker are more representative of the general environment in China.

The recordings were conducted in a soundproof room with ambient noise levels below 30 dB. The recording equipment used was Sound Forge 9.0, with the following software parameters: single-channel, 16-bit depth, and a sampling frequency of 44,100 Hz.

In this study, three pitch levels were operationally defined: “Normal,” “High,” and “Low.” “Normal” pitch was designed to simulate the typical, neutral speaking voice of an adult female in a calm, declarative context, serving as an acoustic baseline. “High” pitch was designed to mimic the elevated pitch register characteristic of CDS, which is known to be more attention-grabbing and perceptually salient. “Low” pitch was included to test perception of a register below the typical conversational range, exploring the boundaries of pitch perception. These three pitch levels were acoustically defined by their relative mean fundamental frequency (F_0_), with validation provided in the acoustic parameter analysis (see Supplementary Data Sheet 1).

To ensure natural vocal quality and prosodic variation, each sentence was recorded three times, each at a different pitch level. The three pitch levels were created via explicit instruction to the talker during the live recording session. The female talker was instructed to produce each sentence using: (1) her natural, neutral speaking voice (“Normal”); (2) a consciously elevated and more animated voice (“High”); and (3) a deliberately lowered and calm voice (“Low”). No post-hoc acoustic manipulation (e.g., pitch shifting) was applied to generate these levels; the different F_0_s were produced natively by the speaker.

For the recording process, Adobe Audition CS6, a professional audio processing software, was employed to reduce noise in each recording. Each sentence was then separated into individual test items for storage, and all files were organized into a central folder for easy access.

Eight graduate students majoring in Speech-Language Pathology and Auditory Rehabilitation, all native Mandarin speakers, conducted the perceptual screening. Following a standardized training and practice session to calibrate their judgments of the three target pitch levels, they independently assessed the three recordings of each sentence. Their task was to independently identify, for each target pitch level (normal, high, low), which of the three recordings was perceived as the clearest and most representative exemplar of that pitch level, considering the naturalness of the production and the perceptual salience of the intended pitch. The recording that achieved the highest consensus among listeners for a given pitch level and sentence was selected for inclusion in the formal test battery.

#### Experimental procedure

Using E-prime 3.0 software, the final set of 30 sentences was integrated into the system and divided into two parts: practice and formal test. Given the young age of the participants and their limited ability to understand written instructions, the experimenter verbally explained the procedure during the practice phase. The practice session began by playing the normal pitch, high pitch, and low pitch versions of the sentence “河里有小鱼 (hé lǐ yǒu xiǎo yú)” to help the participants discern the differences in pitch. Subsequently, the normal pitch, high pitch, and low pitch versions of the sentence “树上有小鸟 (shù shàng yǒu xiǎo niǎo)” were played separately. After hearing each sentence, participants were asked to identify whether the pitch was normal, high, or low. To ensure that participants understood the task before proceeding to the formal test, a comprehension criterion was implemented. Participants completed a minimum of six practice trials (using stimuli not included in the formal test) and were required to correctly identify the pitch of at least 4 out of 6 (≈67%) of these items. If a participant did not meet this criterion, the experimenter provided additional simplified instruction and demonstration, and the practice block was repeated once. All participants successfully met the comprehension criterion within two practice blocks. Once the participants understood the experimental procedure during the practice phase, the instructor initiated the formal test by pressing a button.

Once the participants understood the experimental procedure during the practice phase, the instructor initiated the formal test by pressing a button.

**Figure 1:**
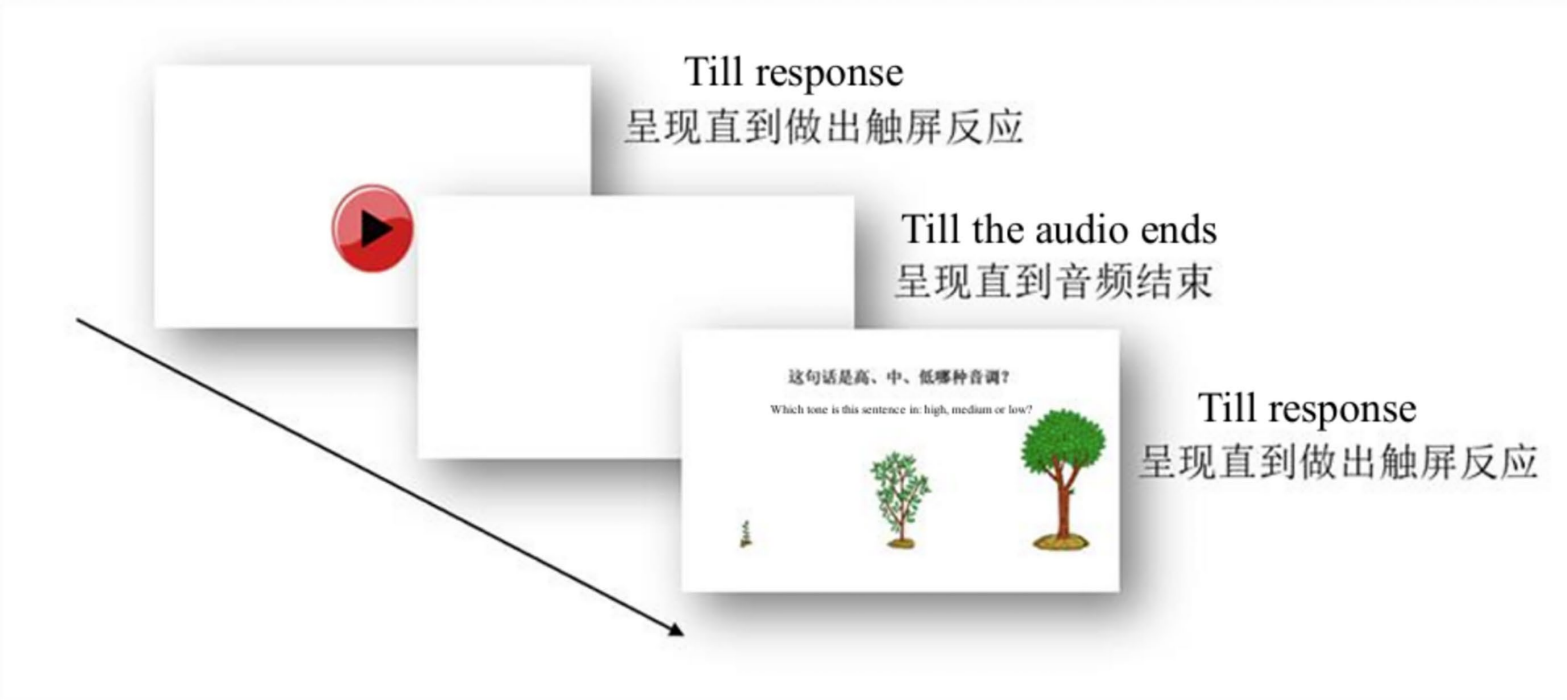
E-prime experiment procedure.

Considering the language and cognitive development levels of children aged 3 to 6 years, the experiment utilized response options in the form of cartoon picture mini-games. Each trial began by the participant tapping a button on the touch screen. After the audio played, three trees of varying heights appeared on the screen: the shortest tree represented the low pitch, the middle tree represented the normal pitch, and the tallest tree represented the high pitch. The positions of the three trees were randomly presented. The child was then asked to determine the pitch and select the corresponding tree. The experimental procedure is illustrated in Figure 1. The entire experiment was conducted using the touch screen, and all results were recorded and saved by E-prime software. Each participant was required to complete 30 tasks. A button was provided for the child to decide whether to pause for a break or continue to the next sentence as needed. The entire experiment took approximately eight minutes to complete.

To clarify the nature of the experimental task, the primary objective was pitch level recognition (identifying whether the target sentence was spoken in high, normal, or low pitch), not lexical tone identification. The factor “Tone type” (T1, T2, T3, T4, Mixed-T) in the analysis refers to the lexical tone context of the sentence in which the target syllable was embedded, rather than a set of categories that participants were explicitly asked to discriminate.

### Data processing

This study employed a 2 × 3 × 5 three-factor mixed experimental design. The independent variables included the type of children (children with CI, NH children), pitch type (normal pitch, low pitch, high pitch), and tone type (T1, T2, T3, T4, Mixed-T), while the dependent variable was the children’s pitch recognition score. A total of 10 sentences (each produced at three pitch levels) yielded 30 individual test items. Each correctly identified item was awarded 1 point, resulting in a maximum possible total score of 30. The scores reported in the Results section (e.g., 17.48 ± 5.29 for NH children) represent summed total scores across all 30 items, allowing for direct interpretation of overall pitch recognition accuracy.

Upon completion of the tests, raw data were extracted from E-prime software and organized. Data were then imported into Microsoft Office Excel, and statistical analysis was conducted using SPSS 26.0. Prior to conducting the repeated-measures ANOVA, Mauchly’s test of sphericity was performed for pitch-related within-subject factors. When the sphericity assumption was violated, the Greenhouse–Geisser correction was applied. For significant main effects and interactions, post-hoc pairwise comparisons were conducted using the Bonferroni correction to control for Type I error.

## Results

### Comparison of pitch recognition characteristics of children with CI and NH children under different tones

#### The effect of pitch recognition of children with CI and NH children at different tones

As shown in Table 3, NH children’s recognition scores for low pitch were higher than those for high and normal pitch. In contrast, children with CI’s recognition scores for normal pitch were lower than those for high and low pitch, although no specific tonal differences were observed. Except for the high pitch in the second tone and the high pitch in the mixed tone, NH children consistently outperformed children with CI in all pitch categories.

**Table 3:** Pitch recognition scores of children with CI and NH children under different tones.

Age	Tone	Pitch	Mean	SD	Age	Tone	Pitch	Mean	SD
NH children	T1	High	1.12	0.78	Children with CI	T1	High	0.84	0.75
		Normal	0.72	0.68			Normal	0.52	0.59
		Low	1.52	0.71			Low	0.88	0.73
	T2	High	1.00	0.76		T2	High	1.00	0.82
		Normal	1.12	0.88			Normal	0.80	0.76
		Low	1.44	0.71			Low	1.00	0.76
	T3	High	0.92	0.81		T3	High	0.88	0.73
		Normal	1.08	0.81			Normal	0.64	0.76
		Low	1.40	0.76			Low	0.64	0.81
	T4	High	1.12	0.83		T4	High	1.04	0.79
		Normal	1.08	0.86			Normal	0.80	0.58
		Low	1.60	0.76			Low	0.72	0.84
	Mixed-T	High	0.72	0.68		Mixed-T	High	0.88	0.73
		Normal	1.20	0.82			Normal	0.64	0.64
		Low	1.44	0.65			Low	0.80	0.87

As indicated in Table 4, the main effect of child type was significant [*F*(1, 48) = 12.39, *p* < 0.001, partial η^2^ = 0.205], and the main effect of pitch was also significant (*F* = 6.198, *p* < 0.01, partial η^2^ = 0.114). However, the main effect of tone was not significant. Regarding interaction effects, the interaction between pitch and child type was significant (*F* = 5.590, *p* < 0.01, partial η^2^ = 0.104), while the interactions between tone and child type, as well as tone and pitch, were not significant. The third-order interaction between pitch, tone, and child type also did not reach significance.

**Table 4:** Analysis of variance about pitch recognition scores of two types of children under different tones.

Variable	df	*F*	*p*	Partial η^2^
Child type	1	12.390	0.001^**^	0.205
Pitch	2	6.198	0.004^**^	0.114
Pitch × Child type	2	5.590	0.007^**^	0.104
Tone	4	1.907	0.126	
Tone × Child type	4	0.301	0.876	
Pitch × Tone	8	1.830	0.099	
Pitch × Tone × Child type	8	0.974	0.470	

^*^p<0.05; ^**^p<0.01; ^***^p<0.001.

To further examine the non-significant effect of lexical tone, we calculated the mean pitch recognition scores (collapsed across high, normal, and low pitch levels) for each tone condition separately for the two groups. For NH children, the mean recognition scores were 1.12 ± 0.52 (T1), 1.19 ± 0.55 (T2), 1.13 ± 0.55 (T3), 1.27 ± 0.58 (T4), and 1.12 ± 0.59 (Mixed-T). For children with CI, the corresponding scores were 0.75 ± 0.53 (T1), 0.93 ± 0.60 (T2), 0.72 ± 0.61 (T3), 0.85 ± 0.59 (T4), and 0.77 ± 0.61 (Mixed-T). These descriptive data confirm the absence of substantial differences across tone conditions within each group, consistent with the non-significant main effect of tone and its interactions. This finding is meaningful as it suggests that the observed pitch recognition deficit in children with CI is not specific to particular lexical tones but rather reflects a domain-general auditory processing difficulty.

The non-significant effect of tone is theoretically meaningful and indicates that the specific Mandarin lexical tone of the sentence (T1–T4 or Mixed-T) did not systematically interfere with or facilitate children’s ability to make global pitch level judgments. This finding helps isolate the observed pitch recognition deficit—particularly the dissociated pattern of preserved high-pitch but impaired normal/low-pitch recognition in children with CI—to a domain-general auditory processing limitation rather than a language-specific phonological deficit.

#### Multiple comparison results and interactions of child types and tones

**Figure 2:**
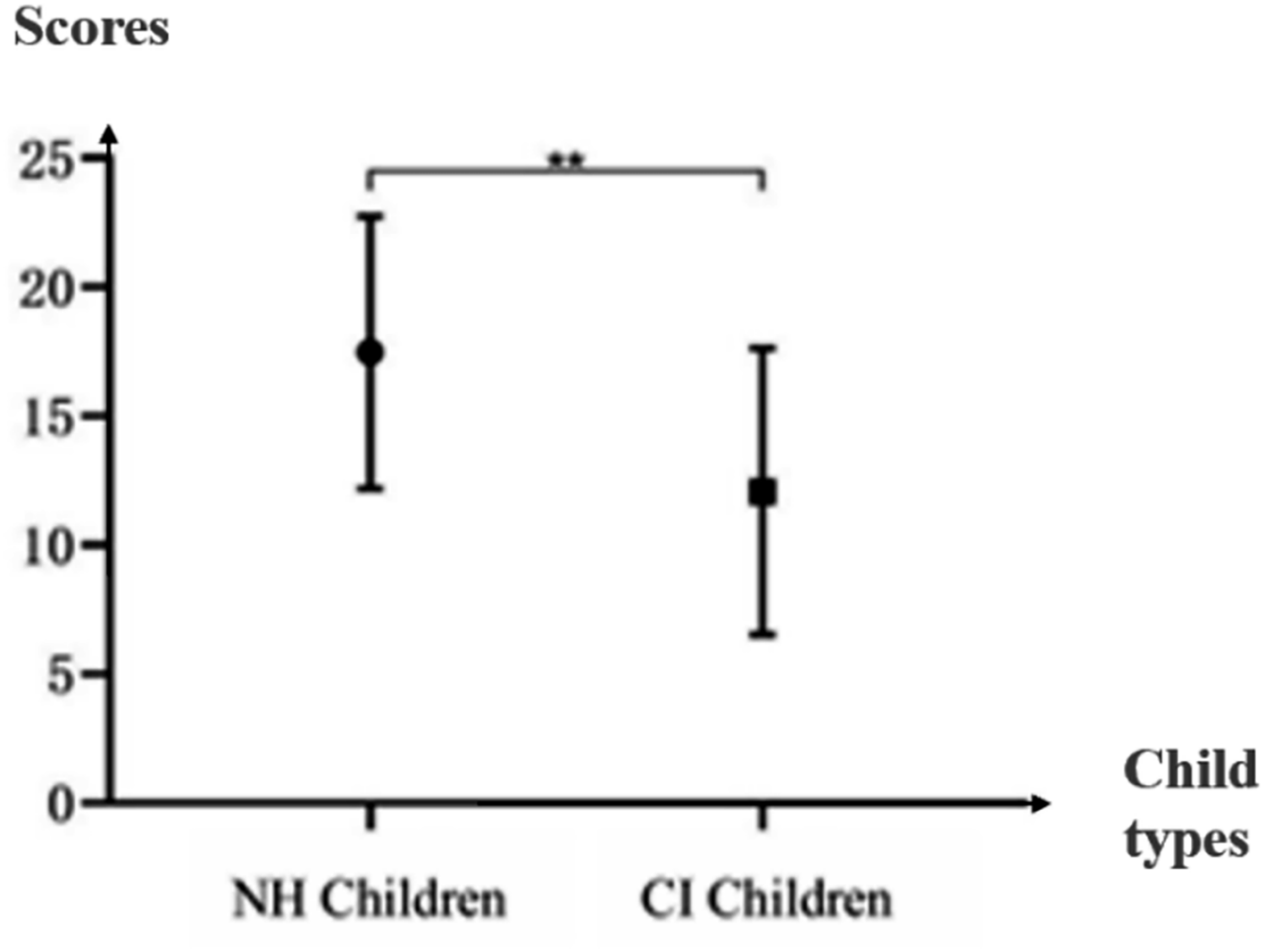
Average pitch recognition scores of children with CI and NH children.

The descriptive statistics for the types of children revealed mean pitch recognition scores of 17.48 ± 5.29 for NH children and 12.08 ± 5.55 for children with CI, as shown in Figure 2. There was a significant difference in pitch recognition scores between children with CI and NH children (*p* = 0.001, <0.01), with NH children demonstrating substantially higher pitch recognition abilities than children with CI.

**Figure 3:**
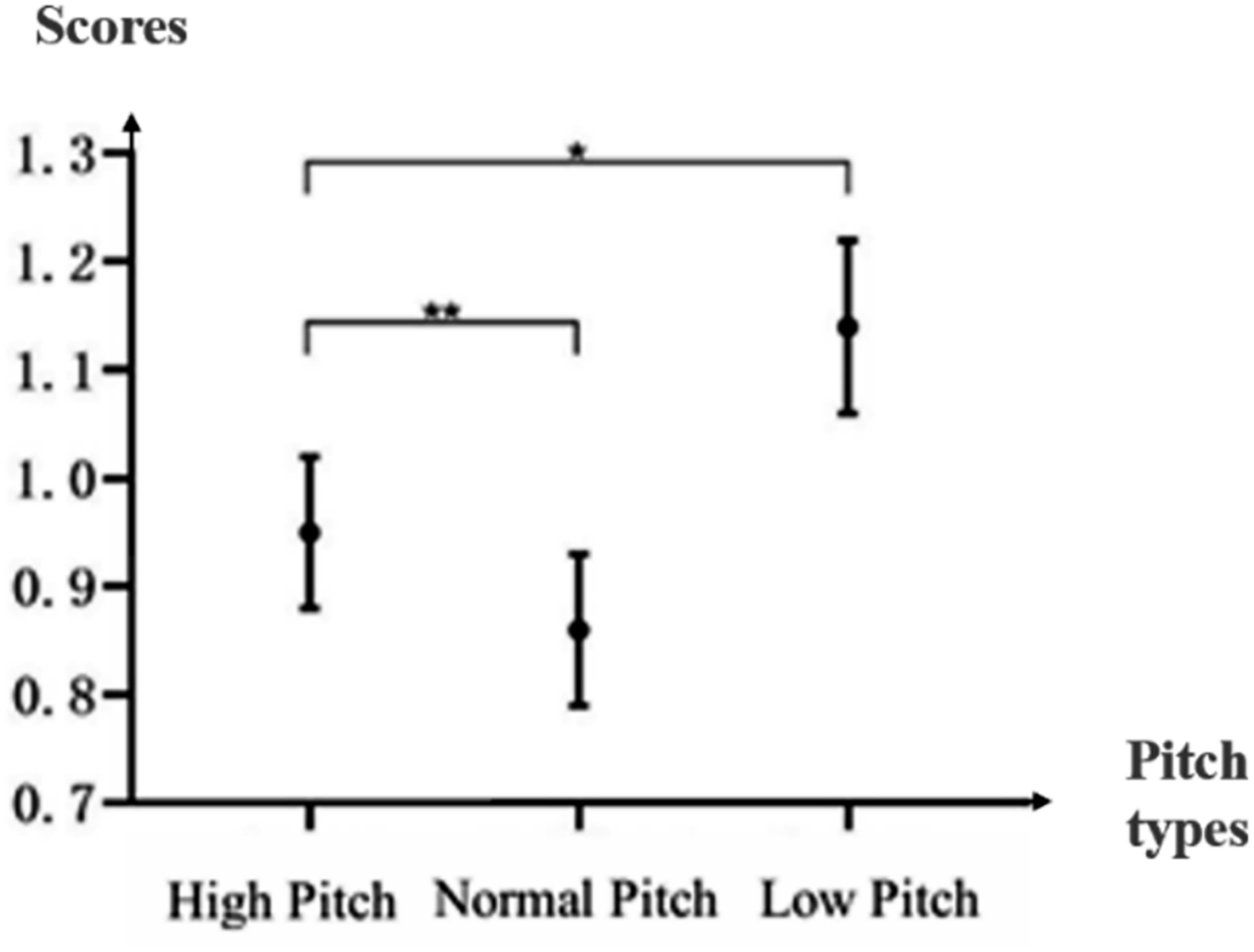
Mean recognition scores for high, normal, and low pitch levels (data combined across CI and NH groups).

Collapsing across both participant groups, the descriptive statistics for pitch showed that the mean recognition scores for high pitch, normal pitch, and low pitch were 0.95 ± 0.07, 0.86 ± 0.07, and 1.14 ± 0.08, respectively, as shown in Figure 3. Supplementary Table 1 presents the inter-group differences in recognition scores for these three pitch categories, based on data combined across the CI and NH groups. It can be seen from Supplementary Table 1 that children’s recognition scores for low pitch were significantly higher than those for high pitch (*p* = 0.048, <0.05) and normal pitch (*p* = 0.001, <0.01). No significant difference was found between recognition scores for high pitch and normal pitch (*p* > 0.05), indicating that across both groups, children exhibited the strongest recognition ability for low pitch.

As can be seen from Supplementary Table 1, children’s recognition scores for low tones were significantly higher than those for high tones (*p* = 0.048 < 0.05) and normal tones (*p* = 0.001 < 0.01). The recognition scores of children for high tones and normal tones did not show a statistically significant difference (*p* > 0.05), from which it can be found that children have the strongest recognition ability for low tones.

**Figure 4:**
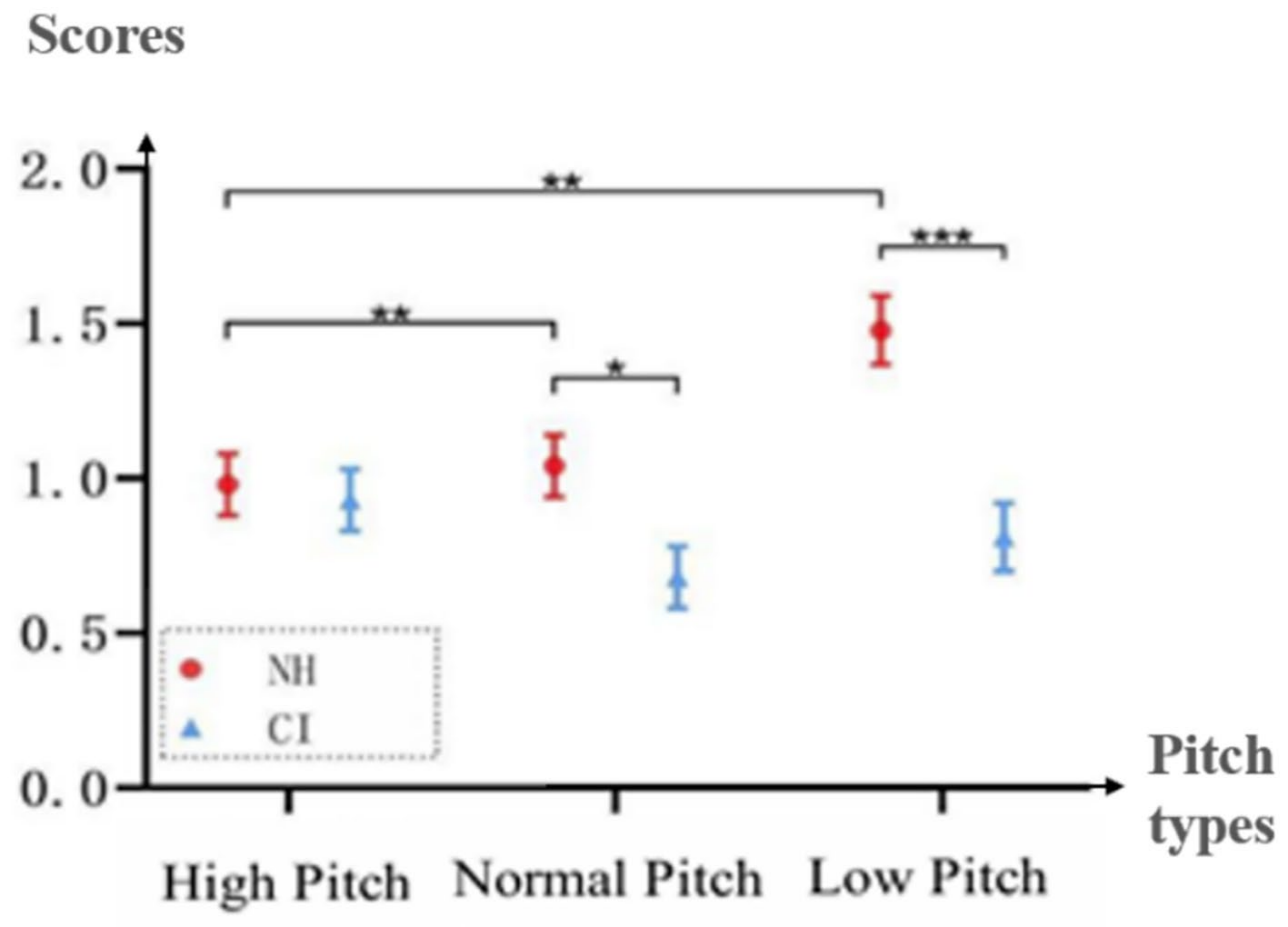
Interaction about pitch × types of children.

The results of the simple effect test, shown in Figure 4, reveal significant differences between NH children and children with CI in recognizing normal pitch (*p* = 0.012) and low pitch (*p* = 0.001). Specifically, NH children showed significant differences in recognition scores for high pitch (*p* = 0.001) and normal pitch (*p* = 0.001) compared to low pitch, while no significant difference was found in children with CI’s recognition scores across these pitches.

### Confusion matrix of pitch recognition of children with CI and NH children under different tones

**Figure 5:**
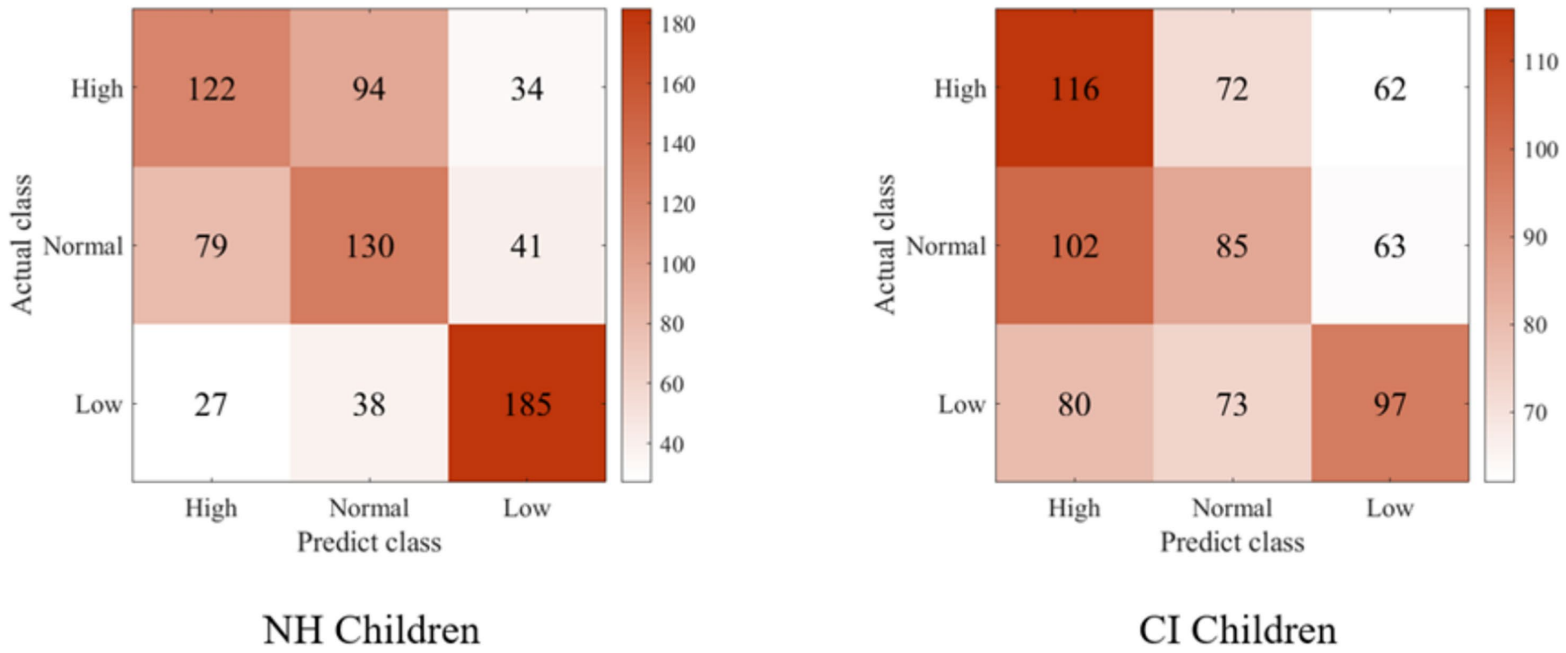
Confusion matrix of pitch recognition in children with CI and NH children (values represent the proportion of responses).

MATLAB R2022b software was used to generate the corresponding confusion matrix based on the pitch recognition scores of children with CI and NH children. The confusion matrix presented in Figure 5 reveals that NH children have better recognition of low pitch, whereas children with CI show a better recognition of high pitch. In NH children, pitch recognition errors were primarily concentrated between high pitch and normal pitch. In contrast, children with CI’s recognition errors were mainly observed between normal pitch and low pitch, with normal pitch being predominantly misidentified as high pitch. Overall, the degree of confusion in pitch recognition was significantly lower in NH children compared to children with CI.

Given the documented importance of age at implantation, we note that the observed dissociated deficit pattern (comparable high-pitch performance but impaired normal and low pitch recognition) was evident across the range of implantation ages present in our sample.

## Discussion

The present study investigated pitch recognition in a cohort of Mandarin-speaking children with CI that, by design, reflects the heterogeneity of the real-world clinical population. This sample included children with different hearing configurations (unilateral, bilateral, and bimodal), varying durations of CI experience (from 6 months upwards), and a wide range of ages at implantation (1.9 to 5.5 years). While such variability introduces complexity, it also provides a robust foundation for examining the core research question: how device-inherent constraints and linguistic factors shape pitch perception in everyday listening contexts. As all children were tested in their everyday listening configuration to maximize ecological validity, the following discussion interprets the findings in light of this heterogeneity, arguing that the consistency of the observed effects across diverse participant characteristics strengthens their generalizability and clinical relevance. The potential influence of limited CI experience, a defining feature of this early-stage cohort, is also explicitly considered.

### Heterogeneity in hearing configuration and the role of bimodal fitting

A key methodological consideration is the inclusion of participants with varying hearing configurations, particularly the four bimodal users (CI + hearing aid). As noted in the introduction, differences in device fitting parameters, such as frequency allocation and signal processing strategies, can influence pitch and tone perception. In this study, we did not statistically “control” for this variability in the traditional sense by treating configuration as a covariate or grouping factor, primarily due to the limited and uneven subsample sizes. Instead, we addressed this issue through our testing methodology and interpretive framing. First, all children were tested in their individual, everyday listening configuration, with no device settings altered for the purpose of the experiment. This approach prioritizes ecological validity, assessing auditory performance as it occurs in the child’s natural environment. Second, and critically, the core finding of the study—a dissociated deficit wherein children with CI performed comparably to NH peers for high pitch but were significantly impaired for normal and low pitch—was observed consistently across the heterogeneous sample. This consistency suggests that the difficulty in processing non-salient, spectrally complex F_0_ information is a fundamental challenge linked to the core sound encoding strategies of current CI systems. Descriptively, this pattern suggests a possible bottleneck that may persist regardless of whether a child uses a unilateral, bilateral, or bimodal configuration, and despite the potential variations in fitting parameters across individuals. However, it should be noted that this observation is based on descriptive inspection rather than formal statistical comparison across configuration subgroups, as the sample sizes within each subgroup were not sufficiently powered for such subgroup analyses. Therefore, rather than viewing the heterogeneity in hearing configuration as a confounding factor to be eliminated, we interpret it as a defining feature of the clinical population. The robustness of our primary finding across this variability strengthens the conclusion that the observed pitch perception deficit is a pervasive issue in pediatric CI users.

### The role of age at implantation

A further source of variability within our CI cohort is the wide range in age at implantation, from 1.9 to 5.5 years. Age at implantation is a well-established factor influencing auditory and linguistic outcomes, as it is closely linked to the timing of auditory input during critical periods of neural plasticity. Consequently, this variability could, in principle, contribute to individual differences in pitch perception performance. We acknowledge this as an important consideration. However, similar to our approach with hearing configuration, we interpret this heterogeneity not merely as a confounding factor, but as a reflection of the real-world clinical reality, where children receive implants at different ages due to diverse etiological and diagnostic timelines. Crucially, our core finding—a dissociated deficit with preserved high-pitch but impaired normal and low-pitch recognition—was observed consistently across this cohort, despite the wide range in implantation ages. This consistency strengthens the argument that the observed deficit is a robust and generalizable phenomenon in the pediatric CI population. It suggests that the fundamental challenge in processing non-salient, spectrally complex F_0_ cues is a pervasive issue linked to the core technological constraints of current CI systems, a challenge that manifests regardless of whether auditory input via the implant began in early toddlerhood or later in the preschool years. Nevertheless, the variability in our sample highlights the need for future research with larger sample sizes to statistically model the specific contributions of age at implantation and duration of device use to the development of pitch perception. Longitudinal studies that track these abilities from the point of implantation are essential to disentangle the effects of auditory experience and neural plasticity from the foundational constraints imposed by CI technology.

### The pitch recognition of children with CI was inferior to that of NH children

The results of this study delineate a distinct pattern of pitch recognition in children with CI: their performance is comparable to NH children for high pitch but significantly impaired for normal and low pitch. This dissociated deficit pattern is consistent with our exploratory hypothesis (H2).

Specifically, the relatively preserved ability to recognize high pitch may be attributed to the overall acoustic salience of the exaggerated, child-directed-speech-like high register and the cues it provides. Such high-pitch sounds may offer amplitude modulation information that CI can transmit with reasonable fidelity (Xu and Pfingst, 2008; See et al., 2013), thereby supporting pitch discrimination in this condition.

In contrast, the significant deficit observed for normal and low pitch is a direct consequence of the CI’s severely degraded encoding of temporal fine-structure (TFS) information, which is critically important for discriminating these pitch levels (Lorenzi et al., 2006; Moore, 2008). Due to the signal processing strategies of current CI—which extract and encode the temporal envelope while discarding fine-structure details—TFS cues are poorly preserved across all frequencies. This limitation is especially consequential for normal and low pitch, where reliance on TFS is greatest.

Thus, the dissociated pattern is best explained by the interaction between acoustic salience and technological constraints, as outlined in H2: high pitch benefits from envelope-coded cues in an acoustically salient register, whereas normal and low pitch suffer from the CI’s inherent inability to represent the fine spectro-temporal details required for accurate perception.

Conversely, the significant deficits in recognizing normal and low pitch are a direct consequence of the device’s limited ability to faithfully represent finer F_0_ variations, which are critical for distinguishing these pitches (Zheng and Brette, 2017). CI processors struggle to provide accurate spectral fine structure information, especially in the lower frequencies. This results in a degraded and spectrally coarser signal, making the subtle differences between normal and low pitch particularly challenging to perceive (Grantham et al., 2022; Wang et al., 2023). This explanation is more consistent with the core technological constraint than the previously proposed attention-based hypothesis.

Furthermore, while child-directed speech is characterized by exaggerated high pitch, which may provide a perceptual advantage for that specific range, the key issue stems from the device’s inherent inability to accurately resolve the temporal fine-structure cues essential for normal and low pitch recognition, a core constraint highlighted in our refined hypothesis (H2). Therefore, the observed dissociation is best explained by an interaction between the acoustic saliency of different pitch levels and the technological bottleneck of current CI systems in processing non-salient, spectrally complex F_0_ information.

Kang et al. (2009) observed in their study on frequency recognition in CI users that they demonstrated higher accuracy in recognizing high-frequency sounds compared to low-frequency ones. This discrepancy can be attributed to CI users’ limited ability to effectively process temporal fine structure cues, which are essential for distinguishing pitch in the mid-to-low frequency range. Furthermore, accurate F_0_ recognition is heavily constrained by the limited number of spectral channels provided by CI. This hardware limitation, inherent in most contemporary implant systems, fundamentally restricts the device’s capacity for precise F_0_ encoding. Underlying organic factors, such as congenital cochlear malformations or ossification, can further compromise low-frequency hearing, thereby exacerbating pitch recognition deficits (Ching et al., 2006). Therefore, the pitch recognition ability of CI users, particularly within the low-frequency spectrum, is generally weaker than that of NH children, a difference that is most pronounced in low-frequency recognition tasks.

It is noteworthy that this dissociated deficit pattern was observed despite the heterogeneity of the CI group in terms of hearing configuration and duration of device use. This consistency suggests that the difficulty in processing normal and low pitch is a fundamental challenge linked to the core sound encoding strategies of current CI systems, a challenge that persists across different clinical fitting approaches and is evident even in the early stages of auditory experience. The inclusion of children with as little as 6 months of CI experience, while potentially a limitation for assessing fully acclimatized performance, offers a valuable window into the perceptual abilities of children during a critical phase of auditory (re)habilitation. The observed deficits at this early stage underscore the pressing need for intervention strategies designed to support pitch perception from the very beginning of a child’s CI journey. The results thus likely reflect a combination of device-inherent constraints and ongoing auditory plasticity, highlighting the need for future longitudinal studies to track how these abilities develop with prolonged experience.

### Tone does not affect the pitch recognition of children with CI

In this study, no significant main or interaction effects of tone were found, suggesting that tone may not be a critical factor in children’s vocabulary learning at the stage under investigation. This null finding is theoretically meaningful: it helps isolate the observed pitch recognition deficit to a domain-general auditory processing limitation rather than a language-specific phonological deficit. Developmentally, children aged 18 to 24 months show sensitivity to errors in Mandarin tone and vowel pronunciation, and their understanding of tones tends to be shaped by their mastery of the native language, often ignoring or adding tone information (Singh et al., 2014). Children who have not yet entered primary school generally lack knowledge of Chinese tones, and therefore their ability to distinguish Mandarin tones in kindergarten is not as developed as that of adults. However, regardless of whether tone-specific teaching is implemented, the ability to recognize Mandarin tones typically begins to emerge around the age of 6 (Chen et al., 2017). With age and increasing language exposure, children’s ability to distinguish tonal differences gradually improves. The four Mandarin tones, despite being produced with different pitches, do not significantly distort the tonal contour even when produced using different pitches in a sentence. The average fundamental frequency (F_0_) of the four tones in Mandarin remains consistent, with the order T4 > T1 > T2 > T3, and the order of the fundamental frequency range is similarly consistent, i.e., T4 > T3 > T2 > T1 (Liu et al., 2009). This stability in tonal contours is critical for the correct interpretation of lexical meaning at the syllable level.

Research on tone recognition among CI users indicates that their ability to recognize tonal contours is significantly impaired, with their overall tone recognition ability being relatively low. Their perceptual sensitivity to the four tones is generally diminished (He et al., 2016). Similarly, while studies by Peng et al. (2004) and Zhou et al. (2013) suggest that Mandarin-speaking children with CI perform poorest on the distinction between T2 and T3, those studies also reported no significant differences in recognition scores between tones in their findings. Furthermore, some researchers, such as Quam and Swingley (2012), argue that children learning English must identify pitch for marking lexical stress, while children learning Mandarin must learn pitch as part of the tonal system. However, a study involving NH and children with CI in the U.S. and Taiwan, who were exposed to three different F_0_ stimuli and asked to identify the differing pitch, found that children with CI’s pitch sensitivity was significantly impaired compared to NH children, with no significant difference based on language (Deroche et al., 2014). This suggests that the advantages of tonal languages may not necessarily enhance overall pitch recognition abilities. Critically, the absence of a tone effect in the present study—combined with the significant main effect of pitch and the Group × Pitch interaction—indicates that the pervasive deficit in recognizing normal and low pitch manifests consistently across all lexical tone contexts (T1–T4 and Mixed-T). This pattern reinforces our conclusion that the core challenge for children with CI stems from the device’s inherent limitations in encoding fundamental frequency information at a basic auditory level, rather than from a language-specific phonological deficit. In other words, the difficulty is domain-general: it affects pitch perception regardless of the linguistic function that pitch serves. Therefore, irrespective of the language or tone involved, all tonal systems adhere to general prosodic principles, and tone does not affect the pitch recognition abilities of children with CI.

### Task comprehension and performance levels

A final methodological consideration pertains to the young age of the participants and the potential influence of task comprehension on the results. As detailed in the Methods section, a rigorous practice phase with a comprehension criterion (at least 67% correct on practice trials) was implemented to ensure that all children understood the three-alternative forced-choice task before proceeding to the formal test. All participants successfully met this criterion, indicating a basic understanding of the task demands. Nevertheless, the overall accuracy for children with CI was below 50% in several conditions, particularly for normal and low pitch. This raises the question of whether the low scores reflect genuine perceptual limits or residual procedural confusion. We argue that the specific pattern of results—children with CI performing comparably to their NH peers on high pitch but showing significant deficits for normal and low pitch—strongly suggests that the observed deficit is not merely a reflection of global task misunderstanding. If the children were simply guessing or were confused by the procedure, one would expect uniformly poor performance across all pitch levels. Instead, the dissociation indicates that while they could successfully apply the task rules to a perceptually salient cue (high pitch), they struggled specifically with the more challenging normal and low pitch stimuli. However, we acknowledge that the inherent difficulty of the pitch discrimination task, combined with the cognitive demands of the three-alternative forced-choice procedure, may have contributed to the low scores, particularly for the younger or less experienced CI users. The relatively high performance on high pitch suggests that when the cue is clear, the task is manageable. Future studies could build on these findings by employing even more extensive training paradigms, using adaptive threshold procedures, or incorporating alternative response methods (e.g., two-alternative forced-choice designs) to further ensure that measured performance reflects perceptual limits rather than procedural challenges.

## Conclusion

This study systematically delineates a distinct dissociation in pitch recognition abilities among Mandarin-speaking children with CI. This dissociated deficit pattern underscores a key constraint in contemporary CI signal processing: the severe degradation of temporal fine-structure information, which is critical for perceiving normal and low pitch, contrasted with the potential support that acoustically salient, high-register pitch may receive from envelope-based cues that are transmitted with better fidelity. Crucially, the finding that Mandarin lexical tone categories did not modulate this effect indicates that the impairment originates from a general auditory-processing deficit in F_0_ encoding rather than a language-specific phonological deficit. These results collectively highlight that pitch recognition in children with CI is shaped by a critical interaction between device limitations and the ecological properties of their auditory input.

Theoretical and practical implications of these findings are substantial. Theoretically, they reframe the challenge of prosodic development in children with CI as not merely a matter of auditory access but of differential access across the pitch spectrum, necessitating models that account for this selectivity. Practically, these results compel a shift in rehabilitative paradigms toward targeted interventions that explicitly train discrimination of normal and low-pitch cues—critical yet underrepresented components for perceiving natural speech prosody. Future research must validate these patterns in larger, longitudinal cohorts and explore innovative avenues such as cross-modal (e.g., visual-tactile) pitch training to circumvent auditory limitations. Ultimately, this work establishes an empirical foundation for developing more nuanced, effective strategies to enhance communicative outcomes in pediatric CI users.

## Data Availability

The raw data supporting the conclusions of this article will be made available by the authors, without undue reservation.
