# Supplementary material for: A comparative study of pitch recognition in children with cochlear implants and normal hearing peers across Mandarin tones
Source: Front Psychol. 2026 Jul 2;17:1783243. doi: 10.3389/fpsyg.2026.1783243 (PMC13373053; doi:10.3389/fpsyg.2026.1783243)
Supplement: Supplementary file 1 [file Data_Sheet_1.docx]

**Acoustic parameter analysis of corpus**

To ensure the selected corpus accurately represents the characteristics of normal, high, and low pitches, Dr.Speech™ software, a speech rehabilitation measuring instrument (Kim et al., 2019), is used to analyze the acoustic parameters of these selected corpus in detail. The fundamental frequency (F_0_), fundamental frequency standard deviation (F_0_SD), was used to analyze the acoustic parameters of the corpus in detail. The analysis provided values for the fundamental frequency (F_0_), fundamental frequency standard deviation (F_0_SD), fundamental frequency range (Range F_0_), speech rate, and mean speech intensity for each sentence. The results of this analysis are presented in Table 1.

According to the normative average speech fundamental frequency for Chinese, the average fundamental frequency for female adults is 230 Hz, with a standard deviation range of ±24 Hz for a single fundamental frequency and ±48 Hz for two fundamental frequencies (Huang, Zhu, & Lu, 2017). Therefore, the speech fundamental frequencies of the three pitch levels recorded in this study align with the normative range for normal speech, without any disturbance to voice quality. These frequencies also fall within the normal pitch, high pitch, and low pitch ranges, and are slightly broader than the fundamental frequency deviation range specified by the norm.

The analysis of the acoustic parameters of the test materials (as shown in Table 1) revealed significant differences in F_0_ and F_0_SD across normal pitch, high pitch, and low pitch (p < 0.05), indicating that the primary acoustic parameters responsible for pitch variation were F_0_ and F_0_SD. This finding is consistent with previous studies (Masataka, 1992). Longitudinal studies across different languages, such as Dravidian, Tagalog, and Korean, have shown that child-directed speech tends to be significantly slower than ADS (Ko, 2012; Narayan & McDermott, 2016). To control for this variable, speech rate was monitored in the current study. The analysis also revealed no significant difference in average speech amplitude between the three pitch levels. Although there were noticeable variations in individual speech speeds, these differences did not impact the children's ability to recognize pitch. Overall, these findings suggest that the corpus used in this study is consistent with the acoustic characteristics of normal pitch, high pitch, and low pitch.

**Table 1.** Acoustic parameter feature analysis of test corpus.

| **Tone** | **Test material** | **F_0_ (Hz)** | | | **F_0_SD (Hz)** | | | **RangeF_0_ (Hz)** | | | **Rate (s)** | | | **Mean Speech Intensity (dB)** | | |
| --- | --- | --- | --- | --- | --- | --- | --- | --- | --- | --- | --- | --- | --- | --- | --- | --- |
|  |  | **high pitch** | **normal pitch** | **low pitch** | **high pitch** | **normal pitch** | **low pitch** | **high pitch** | **normal pitch** | **low pitch** | **high pitch** | **normal pitch** | **low pitch** | **high pitch** | **normal pitch** | **low pitch** |
| T1 | gē gē chī xī guā 哥哥吃西瓜 | 356 | 219 | 192 | 37 | 30 | 22 | 282-430 | 159-279 | 148-236 | 0.314 | 0.3 | 0.324 | 70 | 68 | 70 |
|  | mā mā chuān xīn yī 妈妈穿新衣 | 366 | 249 | 185 | 37 | 47 | 15 | 292-440 | 155-343 | 155-215 | 0.316 | 0.336 | 0.34 | 72 | 71 | 70 |
| T2 | wáng huá laí bá hé 王华来拔河 | 339 | 245 | 173 | 31 | 25 | 17 | 277-401 | 195-295 | 139-207 | 0.294 | 0.338 | 0.29 | 70 | 69 | 71 |
|  | yáng yáng xué lán qíu 阳阳学篮球 | 348 | 214 | 183 | 26 | 21 | 18 | 296-400 | 172-256 | 147-219 | 0.296 | 0.264 | 0.306 | 72 | 71 | 69 |
| T3 | xǐao bǎo dǎ yǔ sǎn 小宝打雨伞 | 294 | 229 | 174 | 48 | 42 | 24 | 198-390 | 145-313 | 126-222 | 0.378 | 0.304 | 0.348 | 66 | 66 | 70 |
|  | xiǎo měi shǔ shǒu zhǐ 小美数手指 | 314 | 215 | 186 | 49 | 40 | 22 | 216-412 | 135-295 | 142-230 | 0.36 | 0.312 | 0.302 | 72 | 73 | 69 |
| T4 | dà xiàng shì dòng wù 大象是动物 | 347 | 271 | 166 | 66 | 56 | 20 | 215-479 | 159-383 | 126-206 | 0.36 | 0.328 | 0.348 | 72 | 71 | 71 |
|  | meì meì qù xùn liàn 妹妹去训练 | 343 | 215 | 177 | 53 | 47 | 24 | 237-449 | 121-309 | 129-225 | 0.302 | 0.27 | 0.356 | 70 | 69 | 71 |
| Mixed-T | xiǎo huá fàng fēng zhēng 小华放风筝 | 336 | 242 | 184 | 72 | 67 | 35 | 192-480 | 108-376 | 114-254 | 0.356 | 0.292 | 0.354 | 66 | 67 | 70 |
|  | gōng yuán yǒu xiǎo shù 公园有小树 | 329 | 227 | 195 | 83 | 37 | 37 | 163-495 | 153-301 | 121-269 | 0.31 | 0.334 | 0.364 | 68 | 72 | 70 |

Note：*Rate(s)* $=\frac{Syllable duration(s)}{The number of syllables}$*.*

According to the normative average speech fundamental frequency for Chinese, the average fundamental frequency for female adults is 230 Hz, with a standard deviation range of ±24 Hz for a single fundamental frequency and ±48 Hz for two fundamental frequencies (Z. M. Huang et al., 2017). Therefore, the speech fundamental frequencies of the three pitch levels recorded in this study align with the normative range for normal speech, without any disturbance to voice quality. These frequencies also fall within the normal pitch, high pitch, and low pitch ranges, and are slightly broader than the fundamental frequency deviation range specified by the norm.

The analysis of the acoustic parameters of the test materials (as shown in Table 2) revealed significant differences in F_0_ and F_0_SD across normal pitch, high pitch, and low pitch (p < 0.05), indicating that the primary acoustic parameters responsible for pitch variation were F_0_ and F_0_SD. This finding is consistent with previous studies (Masataka, 1992). Longitudinal studies across different languages, such as Dravidian, Tagalog, and Korean, have shown that child-directed speech tends to be significantly slower than ASD (Ko, 2012; Narayan & McDermott, 2016). To control for this variable, speech rate was monitored in the current study. The analysis also revealed no significant difference in average speech amplitude between the three pitch levels. Although there were noticeable variations in individual speech speeds, these differences did not impact the children's ability to recognize pitch. Overall, these findings suggest that the corpus used in this study is consistent with the acoustic characteristics of normal pitch, high pitch, and low pitch.

**Table 2.** Paired sample testing of test materials.

|  |  | **t** | **p** |
| --- | --- | --- | --- |
| F_0_ | high pitch-normal pitch | 16.16 | .001^**^ |
|  | high pitch-low pitch | 25.46 | .001^**^ |
|  | normal pitch-low pitch | 6.96 | .001^**^ |
| F_0_SD | high pitch-normal pitch | 2.23 | .05^*^ |
|  | high pitch-low pitch | 7.02 | .001^**^ |
|  | normal pitch-low pitch | 4.06 | .001^**^ |
| Rate | high pitch-normal pitch | 2.33 | .04^*^ |
|  | high pitch-low pitch | 0.10 | .92 |
|  | normal pitch-low pitch | -2.40 | .04^*^ |
| Mean Speech Intensity | high pitch-normal pitch | 0.00 | 1.00 |
|  | high pitch-low pitch | -1.01 | .33 |
|  | normal pitch-low pitch | -0.96 | .36 |
