# Supplementary figures and images for: A comparative study of pitch recognition in children with cochlear implants and normal hearing peers across Mandarin tones
Source: Front Psychol. 2026 Jul 2;17:1783243. doi: 10.3389/fpsyg.2026.1783243 (PMC13373053; doi:10.3389/fpsyg.2026.1783243)

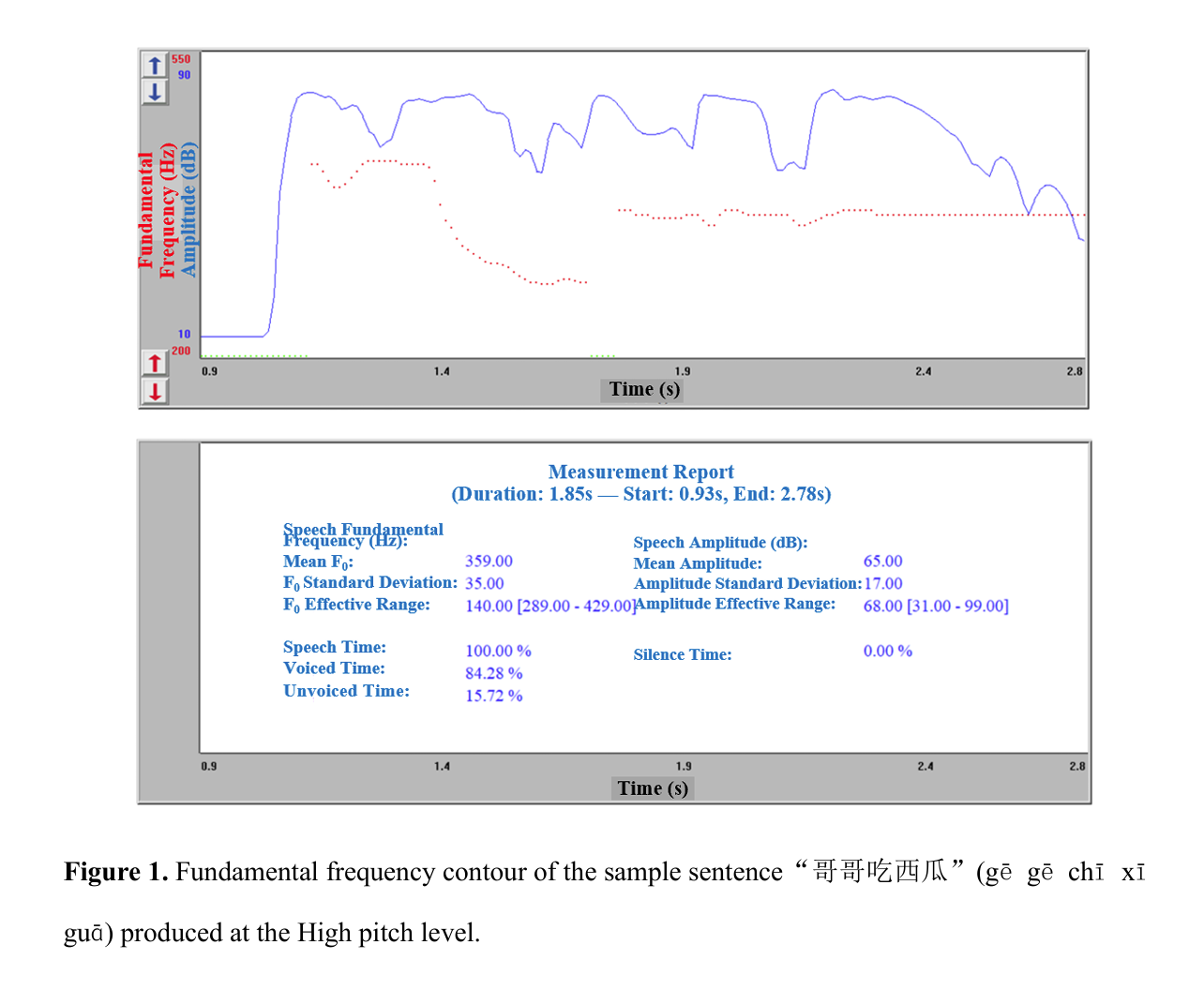

Supplement: Supplementary file 2 [file Image_1.png]

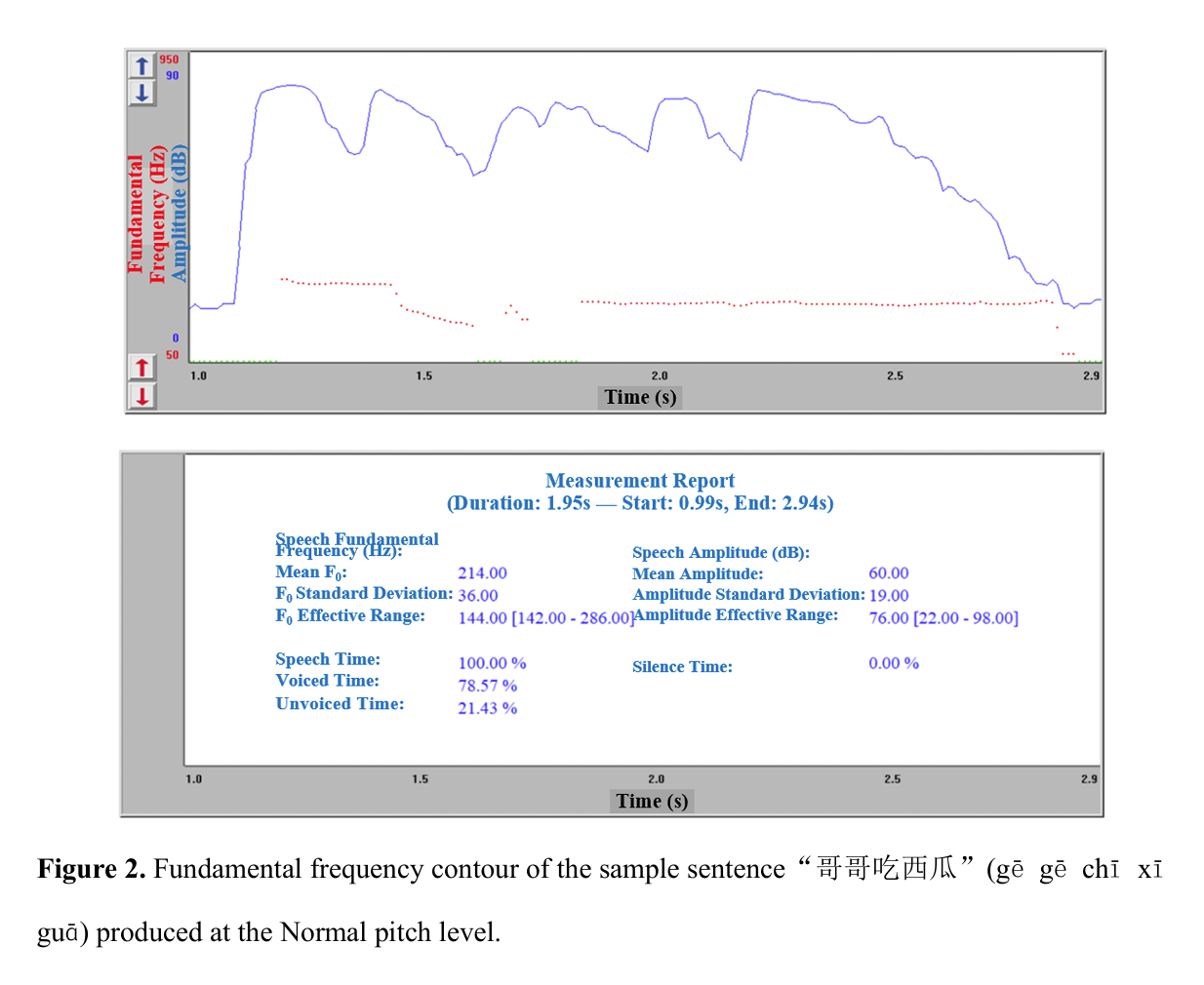

Supplement: Supplementary file 3 [file Image_2.png]

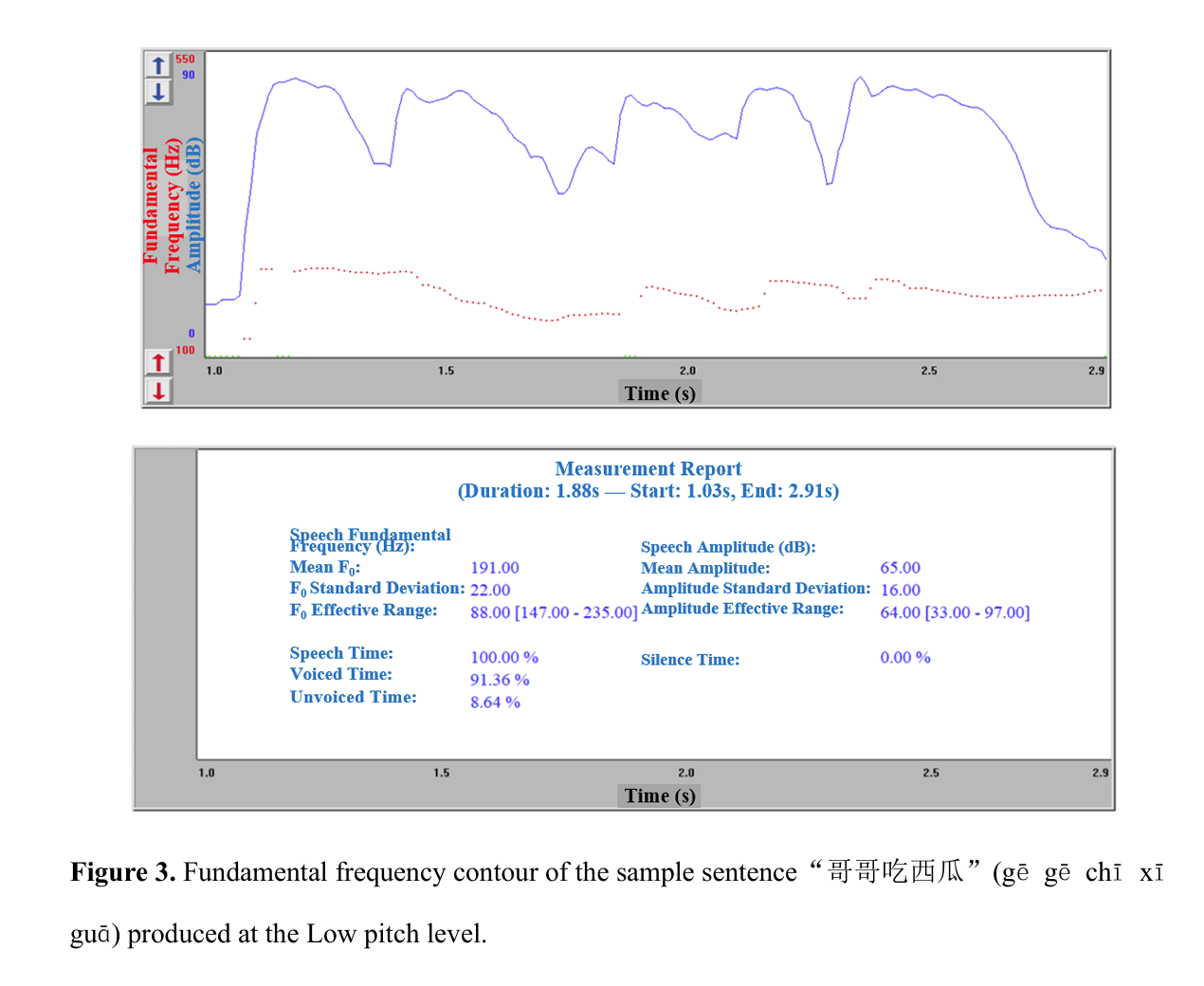

Supplement: Supplementary file 4 [file Image_3.png]
