# Supplementary material for: A comparative study of pitch recognition in children with cochlear implants and normal hearing peers across Mandarin tones
Source: Front Psychol. 2026 Jul 2;17:1783243. doi: 10.3389/fpsyg.2026.1783243 (PMC13373053; doi:10.3389/fpsyg.2026.1783243)
Supplement: Supplementary file 5 [file Table_1.docx]

**Supplementary Table 1.** Results of multiple comparisons of recognition scores of high pitch, normal pitch and low pitch.

| **pitch** | **pitch** | ***p*** |
| --- | --- | --- |
| high | normal | .363 |
|  | low | .048^*^ |
| normal | high | .363 |
|  | low | .001^**^ |
| low | high | .048^*^ |
|  | normal | .001^**^ |

Note：*p*^*^＜0.05; *p*^**^＜0.01
